# Synthesis of ZnO Nanoparticles Loaded on Biochar Derived from *Spartina alterniflora* with Superior Photocatalytic Degradation Performance

**DOI:** 10.3390/nano11102479

**Published:** 2021-09-23

**Authors:** Hua Jing, Lili Ji, Zhen Wang, Jian Guo, Shiyao Lu, Jiaxing Sun, Lu Cai, Yaning Wang

**Affiliations:** 1 National Marine Facilities Aquaculture Engineering Technology Research Center, Zhejiang Ocean University, Zhoushan 316022, China; jinghua20190217@163.com (H.J.); wz517247442@163.com (S.L.); sjx1686078390@163.com (J.S.); wyn198399@126.com (Y.W.); 2 Zhejiang Lichen New Material Technology Co., Ltd., Hangzhou 310000, China; www.wzzjou@163.com; 3 College of Food and Medical, Zhejiang Ocean University, Zhoushan 316022, China; guojian@zjou.edu.cn; 4 Donghai Science and Technology College, Zhejiang Ocean University, Zhoushan 316000, China; cailuyxy@zjou.edu.cn

**Keywords:** zinc oxide, green synthesis, *Spartina alterniflora*, composite photocatalyst, dye degradation mechanism

## Abstract

*Spartina alterniflora* is an invasive plant from coastal wetlands, and its use in applications has garnered much interest. In this study, a composite photocatalyst (ZnO@BC) was synthesized by preparing zinc oxide (ZnO) nanoparticles with *S. alterniflora* extracts, *S. alterniflora*, and one-step carbonization, which was characterized using scanning electron microscope (SEM), transmission electron microscopy (TEM), Fourier transform infrared spectroscopy (FT-IR), Raman, X-ray photoelectron spectroscopy, ultraviolet–visible spectroscopy (UV–vis DRS), photoluminescence (PL) and N_2_ adsorption–desorption isotherm. The degradation capacity and mechanism of malachite green (MG) using ZnO@BC were analyzed under visible irradiation, and the degradation products of malachite green were detected by LC–MS. The results show that ZnO@BC has a larger surface area (83.2 m^2^/g) and various reactive groups, which enhance its photocatalytic efficiency, with the presence of oxygen vacancy further improving the photocatalytic activity. The total removal rate of malachite green (400 mg/L) using ZnO@BC is up to 98.38%. From the LC–MS analysis, it could be concluded that malachite green is degraded by demethylation, deamination, conjugate structure and benzene ring structure destruction. This study provides a novel idea for the high-value utilization of *S. alterniflora*.

## 1. Introduction

With production rapidly developing across human society, a variety of pollutants increasingly enter the environment [1,2], far exceeding the degradation capacity of the environment itself [3]. Among them are organic dye pollutants, which threaten the environment and human health due to their complex composition, high toxicity and hard-to-degrade nature [4]. As a synthetic triphenylmethane compound, malachite green (MG) is both a dye and a fungicide, widely used across various industries, including the textile and aquaculture industries [5]. While it has a range of side effects on the environment, such as high toxicity, high residue, teratogenicity, and mutagenesis, it is easily absorbed and enriched by organisms [6,7,8]. The treatment of MG has always been the focus of dye wastewater treatment.

Due to traditional treatment methods for dye wastewater being of high cost and ineffective [9], in recent years, various advanced treatment technologies for dye wastewater have been developed, such as electrochemical technology [10], ion exchange [11], biosorption [12], membrane adsorption filtration [13] and photocatalytic degradation [14].

Photocatalytic technology, as an advanced oxidation process (AOP), provides an efficient and environmentally friendly method for the treatment of MG wastewater [15], which can realize the decolorization and mineralization of MG, eventually transforming MG into carbon dioxide and inorganic acid; possesses a low cost; is a simple operation; and has a high degradation efficiency [16].

Semiconductor metal oxides have attracted much attention because of their unique photocatalytic properties. These include zinc oxide (ZnO) as a second-generation photocatalyst, as it is non-toxic, low-cost, stable in nature and environmentally friendly [17], contributing to its higher photoelectric conversion efficiency [18]. Recently, ZnO nanoparticles have attracted extensive attention due to their large specific surface area and high active sites [19,20,21,22]. Meanwhile, ZnO is a multifunctional material, which means that it has many areas of application [23]. Up to now, there have been various preparation methods for ZnO nanoparticles, e.g., physical, chemical and biological methods [24]. Chemical and physical methods include precipitation, microemulsion, chemical reduction, sol-gel and hydrothermal techniques, and pulsed laser deposition [25]. Synthesized ZnO nanoparticles with different sizes and morphologies can play an excellent role in different fields. However, chemical and physical methods generate a higher cost; in particular, secondary metabolic waste can be generated and causes secondary pollution to the environment [26,27]. Biological methods have a wide range of raw materials, including plants, bacteria and algae, among which it is found that ZnO nanoparticles prepared from plant extracts are more stable than those prepared from other raw materials [28] because there are abundant reductive small molecules in plants, such as terpenes, polyphenols, alkaloids, phenolic acids, flavonoids, quinones, etc., which can replace chemical agents as dispersants and stabilizers in the process of synthesizing ZnO nanoparticles [29]. The process of ZnO nanoparticles prepared from plant extracts is mild, environmentally friendly, highly efficient and low-cost and is in line with the direction of social progress [30]. Recently, a variety of plants have been applied for the preparation of ZnO nanoparticles, such as *mint leaves* [31], *Phoenix dactylifera* [32], *Peganum harmala* seed extract [33], *Alchornea laxiflora* leaf extract [34] and *Couroupita guianensis Aubl* leaf extract [35].

*S. alterniflora* is an alien invasive plant in China with strong adaptability, fast reproduction, spreading capabilities and high resistance to salinity [36], which can displace native species, thereby threatening local biodiversity [37] and damaging the natural ecosystems and coastal aquaculture, therefore causing direct economic losses for China [38]. However, *S. alterniflora* as a plant can serve as a resource, and previous studies have reported that the transformation of *S. alterniflora* into biochar by pyrolysis can be applied in the remediation of heavy metal contamination [39,40,41]. Xu et al. [42] found that *S. alterniflora* leaf extracts contain several reducing components and mainly flavonoids. However, there is no report on the synthesis of ZnO nanoparticles with *S. alterniflora* extract and little is known about the mechanism of adsorption and the photocatalytic degradation of dyes on biochar derived from *S. alterniflora*-loaded metal oxide. To the best of our knowledge, there is no report on ZnO nanoparticles using *S. alterniflora* extract.

Hence, in this study, ZnO nanoparticles were synthesized using *S. alterniflora* extract loaded on biochar derived from *S. alterniflora* by a one-step carbonization method and prepared into a ZnO@BC photocatalyst. Scanning electron microscope (SEM), transmission electron microscopy (TEM), Brunauer–Emmett–Teller (BET), Fourier transform infrared spectroscopy (FTIR), X-ray diffractometer (XRD), Raman spectroscopy, X-ray photoelectron spectroscopy (XPS), ultraviolet–visible spectroscopy (UV–vis DRS) and photoluminescence (PL) were used to characterize the physical and chemical properties of ZnO@BC. The photodegradation capacity and mechanism of MG using ZnO@BC were discussed under visible irradiation, and the degradation products of MG were detected by LC–MS.

## 2. Materials and Methods

### 2.1. Materials

*S. alterniflora* leaves used in this experiment were from the tidal flat of Zhoushan, Zhejiang, China; were cleaned with deionized water three times; and were dried in the oven (DGG-9030BD, Shanghai Senxin) overnight at 60 °C. The pretreated *S. alterniflora* was smashed into powder (D ≤ 100 mm) and placed in the dryer.

Malachite green (MG, C_23_H_25_ClN_2_), Zinc oxide (ZnO), zinc acetate dihydrate ((CH_3_COO)_2_Zn·2H_2_O), polyvinylpyrrolidone ((C_6_H_9_NaOH)_n_, K30) and sodium hydroxide (NaOH) were all purchased from Sinopharm Chemical Reagent Co., Ltd. (Shanghai, China). The chemicals applied in the present study were all analytical grade without further treatment.

### 2.2. Synthesis of ZnO Nanoparticles

Two grams of *S. alterniflora* powder were mixed with four hundred milliliters of ethanol in a volumetric flask, placed in a water bath at 80 °C for two hours and centrifuged (TG16-WS, Hunan Xiangyi) at 6000 r/min for one min. The supernatant was taken to obtain the *S. alterniflora* extract; 1.756 g of 0.02 mol/L zinc acetate dihydrate was added into 200 mL distilled water, placed in a magnetic stirrer (ZNCL-GS, Aibot Technology, Honkong, China), and stirred for one min before adding 0.1 g polyvinylpyrrolidone and stirring magnetically for five min; 100 mL of *S. alterniflora* extract was slowly taken into the above mixed solution; and 100 mL of 0.4 mol/L NaOH solution was also gently added, kept at 80 °C and stirred magnetically for 30 min until a pale green precipitate was generated, namely the precursor of ZnO nanoparticles.

### 2.3. Synthesis of ZnO@BC Photocatalyst

A total of 1.756 g of *S. alterniflora* powder was mixed with 400 mL of the precursor of ZnO nanoparticles, placed in a water bath at 80 °C for two hours and centrifuged at 8000 r/min for five min. The precipitate was dried overnight at 80 °C in an oven, carbonized in a tubular furnace under nitrogen flow (200 mL min^−1^), first heated to 200 °C with a rate of 5 °C/min for 30 min and then heated to 800 °C with the same rate for two hours. This cooled mixture was washed three times with deionized water until the pH was neutral, dried in an oven at 60 °C for 24 h and sieved with 120 mesh (ASTM standard). A black powdered photocatalyst from *S. alterniflora*-based zinc oxide photocatalyst was obtained, denoted as ZnO@BC.

### 2.4. Characterization of ZnO@BC Photocatalyst

The morphological and microstructures of the as-prepared samples were analyzed by scanning electron microscopy (SEM, Su8010, Hitachi, Tokyo, Japan) and transmission electron microscopy (TEM, Tecnai G2 F20 S-Twin, FEI, Hillsboro, OR, USA). The as-prepared sample was degassed at 300 °C for two hours, and then, an N_2_ adsorption/desorption isotherm was performed on an automatic specific surface area and pore analyzer (ASAP 2460, Micromeritics, Norcross, GA, USA) and calculated using the method of Brunauer–Emmett–Teller (BET). The X-ray diffraction patterns were recorded on an X-ray Diffractometer (XRD, D/max2500, Rigaku, Japan) in the range of 2θ from 20° to 80°. The functional groups were analyzed by Fourier transform infrared spectroscopy (FTIR, Nicolet 6700, Thermo Scientific, Hillsboro, OR, USA). The surface chemical state was characterized by X-ray photoelectron spectroscopy (XPS, Scientific EscaLab 250Xi, Thermo Fisher, Hillsboro, OR, USA). The light absorption was determined by UV–vis diffuse reflectance spectroscopy (UV–Vis DRS, UV 2600, Shimadzu, Japan). The electron–hole recombination rate was studied by fluorescence emission spectroscopy (PL, FLS980, Edinburgh Instruments, Edinburgh, UK).

### 2.5. Photodegradation of MG Using ZnO@BC Photocatalyst

The degradation experiments on as-prepared samples were conducted by an XPA-7 photochemical reactor (Xujiang Electromechanical Plant, Nanjing, China) by adding 40 mg of as-prepared samples into 100 mL of 400 mg/L MG solution, magnetically stirred in the dark until adsorption–desorption equilibrium was achieved and then illuminated for one hour under a 300 W xenon lamp with a 390 nm cut-off filter (FSX-300, NBeT Group Corp., Beijing, China). An aliquot (20 mL) was collected at 20 min intervals, separated by centrifugation at 8000 rpm for two min. The supernatant was analyzed at a wavelength of 617 nm (MG) with a UV–vis spectrophotometer (UV 2600, Shimadzu, Japan).

The photocatalytic degradation rate was calculated from Equation (1):

(1) $$D=\frac{A_{0}-A_{t}}{A_{0}}\times 100\%$$

where D (%) is the degradation rate of the as-prepared samples, A_0_ is an initial of MG and A_t_ is the absorbance value measured at some reaction time point.

### 2.6. Active Species Detection

Two milliliters of dimethyl sulfoxide (DMSO, 0.1 moL/L), formic acid (FA, 0.1 moL/L) and *p*-benzoquinone (BQ, 0.05 moL/L) as scavengers for •OH, H^+^ and •O^2−^, respectively, were added into the photocatalytic reaction solution before the illumination.

### 2.7. Photocatalytic Degradation Products of MG Analysis

The degradation products of MG using ZnO@BC were detected by liquid chromatography–mass spectrometry (LC–MS, Q-TOF 6540, Agilent, Santa Clara, CA, USA) equipped with an electrospray ionization (ESI) positive ion mode. The test conditions were as follows: the mobile phase includes two solutions, namely A and B. Solution A was made of 0.1 m acetate and acetic acid (pH 5.3), and solution B was acetonitrile. The gradient elution ranged from 5% to 95% in 30 min, with the flow rate of 0.8 mL/min.

## 3. Results

### 3.1. Morphology and Microstructure Characterization

#### 3.1.1. SEM and TEM Analysis

**Figure 1:**
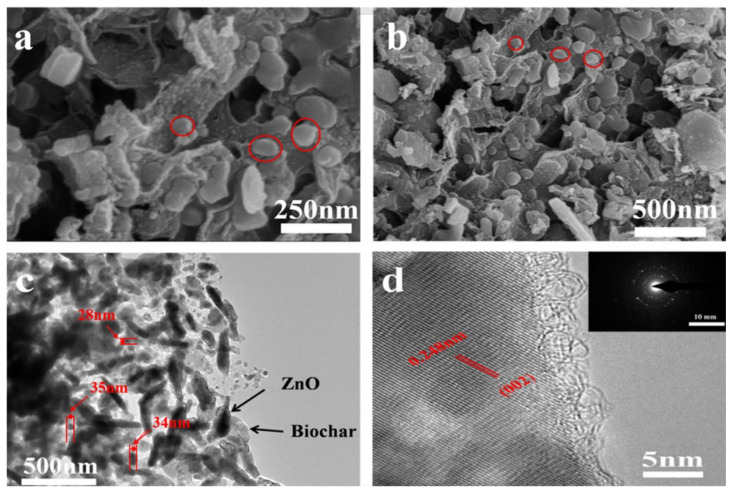
(**a**,**b**) SEM images of ZnO@BC photocatalyst; (**c**,**d**) TEM images of ZnO@BC photocatalyst.

SEM images of the ZnO@BC photocatalyst at different magnifications exhibit its fluffy structure with variable-sized pores, as shown in Figure 1a,b, ZnO particles are well dispersed across the surface of *S. alterniflora* biochar, indicating that green synthesis of ZnO nanoparticles with the *S. alterniflora* extract reduces their agglomeration. Meanwhile, Figure 1c,d shows ZnO@BC nanoparticles TEM images that display near-rodlike morphology and estimate that the size of ZnO@BC is 25–40 nm at the nanoscale, which were wrapped in the *S. alterniflora* biochar, further confirming that ZnO nanoparticles have been successfully loaded on the surface of S. alterniflora biochar. It can be seen from the crystallographic diffraction fringes of HRTEM that the crystal plane spacing of ZnO@BC is 0.248 nm corresponds to the (002) lattice planes of the ZnO crystal.

#### 3.1.2. Porous Structure

**Figure 2:**
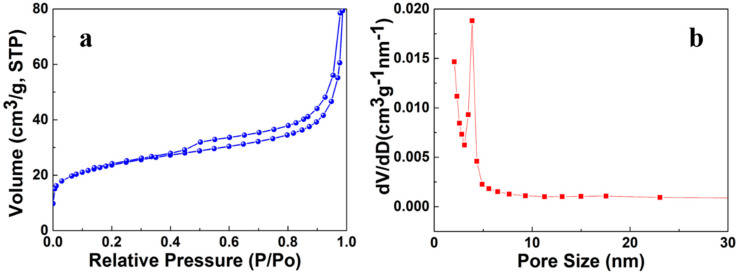
(**a**) N_2_ adsorption-desorption isotherm; (**b**) pore size distribution of ZnO@BC.

According to IUPAC classification [43], it has been found that the N_2_ adsorption isotherm of ZnO@BC is type IV, as shown in Figure 2. In the low P/P_0_ region, the isotherm is convexity upward; in the higher P/P_0_ region, it rises rapidly due to the capillary condensation of mesoporous solid after multi-layer adsorption with a hysteresis loop at P/P_0_ > 0.4, suggesting the presence of mesopores [44]. In addition, it can be seen from the pore size distribution curve that there are concentrated distributions of mesopores and micropores, and the composite adsorption isotherm model. The specific surface area and the pore volume of ZnO@BC analyzed from N_2_ adsorption isotherm data are limited to 83.2 m^2^/g and 0.1233 cm^3^/g. In the synthesis process of ZnO@BC photocatalyst, *S. alterniflora* has been transformed into porous biochar through pyrolysis reaction, which not only provides abundant loaded sites for ZnO particles but also furnishes various adsorption sites for dye molecules.

### 3.2. Chemical Compositions Characterization

#### 3.2.1. XRD Analysis

**Figure 3:**
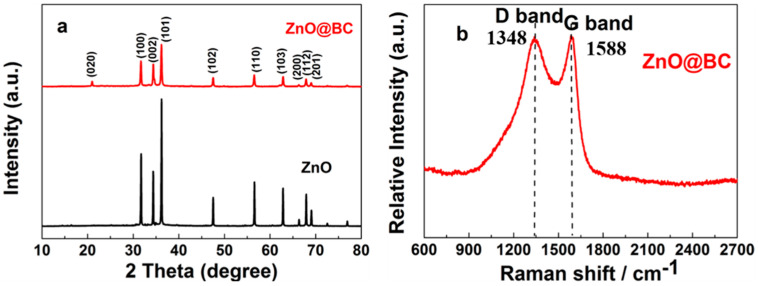
(**a**) XRD patterns of ZnO@BC; (**b**) Raman spectra of ZnO@BC.

The crystal structure and phase analysis of ZnO@BC was performed by XRD, as shown in Figure 3a. The XRD of ZnO@BC show almost similar peak positions but different peak intensities compared with that of ZnO. The peaks located at 31.76°, 34.42°, 36.25°, 47.53°, 56.60°, 62.86°, 66.37°, 67.96° and 69.09° were perfectly indexed to the (100), (002), (101), (102), (110), (103), (200), (112) and (201) planes of hexagonal wurtzite, which were referenced to the JCPDS 36–1451 [45]. However, in the XRD patterns of ZnO@BC, there is a diffraction peak at 2θ = 21.08° corresponding to the (020) plane of cellulose II [46] due to a large amount of crystalline cellulose being formed during the carbonization process of *S. alterniflora*. XRD characterization of the as-prepared sample further demonstrates that ZnO nanoparticles have been successfully prepared by green synthesis and loaded on the *S. alterniflora* biochar.

The crystallites size ZnO@BC was calculated by the Scherrer Equation (2), which is 30.6 nm. Furthermore, it is proven that the theoretical calculation value is close to the actual particle size from the TEM image.

(2) $$D=\frac{K\lambda }{\beta \cos\theta }$$

D is the size of ZnO@BC, K is Scherrer constant value of 0.89, λ is X-ray wavelength of 0.154056 nm, β is diffraction peak half-height width and θ is the Bragg diffraction angle.

#### 3.2.2. Raman Analysis

The Raman spectra (Figure 3b) of the as-prepared samples show that there are two peaks at 1348 cm^−1^ and 1588 cm^−1^, corresponding to the D band and G band, respectively, among which the D band is attributed to sp^3^-hybridized carbon in a disordered state, while the G band is associated with the vibration of sp^2^-hybridized carbon in a graphitic layer [47,48]. The ratio of I_D_/I_G_ is a measure of the disorder of the graphite layers, and the smaller the I_D_/I_G_ ratio, the higher the degree of graphitization. The I_D_/I_G_ ratio of ZnO@BC is 0.99. The graphitization of ZnO@BC is influenced by *S. alterniflora* biochar, with the higher graphitization being attributed to the transfer of electrons during photocatalysis.

#### 3.2.3. FTIR Analysis

**Figure 4:**
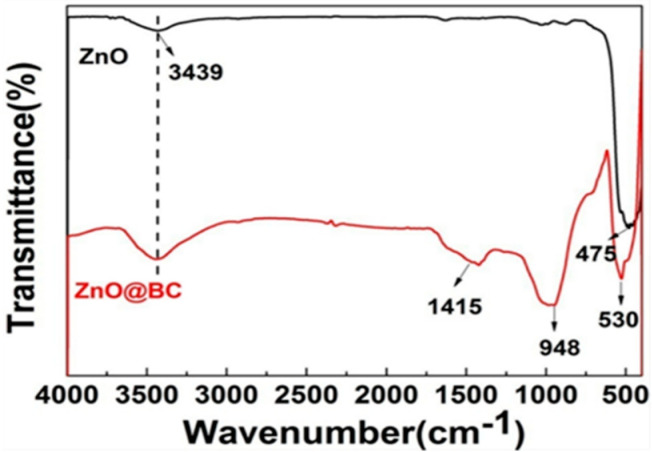
FTIR spectra of ZnO@BC.

The FTIR spectra (Figure 4) of ZnO@BC and ZnO all exhibit a broad band at 3400–3500 cm^−1^ attributed to –OH stretching mode, and a sharp peak at 550–420 cm^−1^ ascribed to the characteristic absorption peak of Zn–O [49]. The absorption peak at 987 cm^−1^ in the ZnO@BC spectrum is attributable to the C–H bending of aromatics, and that at 1415 cm^−1^ is associated with the C=O stretching vibration [50]. The FTIR spectrums of ZnO@BC demonstrate that ZnO nanoparticles have been loaded onto the surface of biochar, which is consistent with its XRD patterns. There are various oxygen-containing functional groups on the surface of ZnO@BC, such as –OH and C=O, serving as potential adsorption sites to adsorb the organic dyes [51].

#### 3.2.4. XPS Analysis

**Figure 5:**
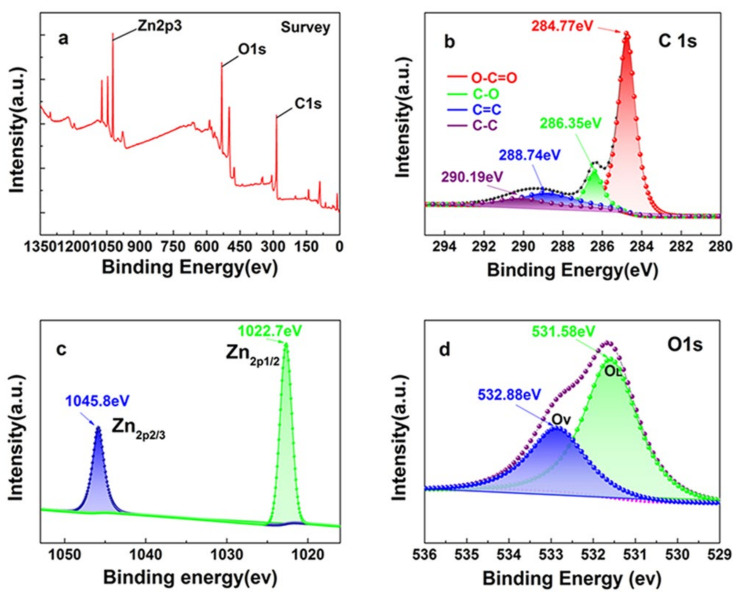
(**a**) XPS survey spectrum of ZnO@BC; (**b**) C 1s; (**c**) Zn 2p_1/2_ and 2p_3/2_; (**d**) O 1s.

The XPS spectra (Figure 5) show that the three sharp peaks at 1024 eV, 532 eV and 285 eV correspond to the characteristic peaks of Zn 2p3, C 1s and O 1s, respectively, demonstrating that ZnO@BC mainly contains C, O and Zn. The peaks in the C 1s spectrum with the relevant binding energies of 290.19 eV, 288.74 eV, 286.35 eV and 284.77 eV, correspond to O-C=O, C-O, C=C and C-C, respectively [52], which could be active sites, enhancing the adsorption capacity of ZnO@BC for organic dyes. The two sharp peaks observed at 1045.8 eV and 1022.7 eV in the Zn 2p3 spectrum corresponded to Zn 2p1/2 and Zn 2p3/2, respectively, which are spin-orbit split components in the form of Zn^2+^ [53]. In the O 1s spectrum, the lower binding energy phase (531.58 eV) is attributed to the oxygen lattice (O_L_) with a hexagonal wurtzite of ZnO, while the higher binding energy phase (532.88 eV) is ascribed to oxygen vacancies (O_V_) on the surface of ZnO@BC. ZnO typically tended toward the formation of surface Zn–OH groups, while Ov is attributed to hydroxyl groups, or chemisorbed or dissociated oxygen on the surface of ZnO@BC [54], where a change in the intensity of Ov-related components could be ascribed to the change in the concentration of surface oxygen defects. The formation of heterojunctions at the surface of ZnO@BC nanoparticles could be responsible for the enhancement in the number of Ov. Ov could promote the charge separation effectively, extend the photo response region of the photocatalyst and provide active sites for the photocatalytic reaction [55].

### 3.3. Semiconductor Performance Characterization

#### 3.3.1. UV–Vis DRS and Energy Band Gap Analysis

**Figure 6:**
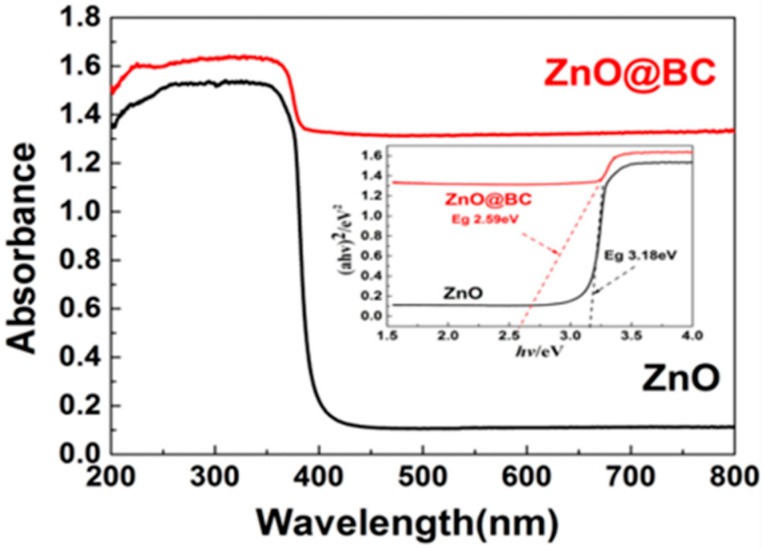
UV-vis spectrum and band gap of ZnO@BC and chemical reagent.

It could be seen that there is a broad characteristic peak at 250–380 nm in the UV–vis spectrum (Figure 6) of ZnO@BC and ZnO, ascribed to the basic bandgap absorption of ZnO [56]. Compared with ZnO, ZnO@BC exhibits an intense absorption in the UV and visible light regions, which is suggest to be induced by the Biomass Carbon content [57]. ZnO@BC has a slight redshift in the adsorption edge and has a calculated band gap (Eg) of 2.59 eV, compared with ZnO’s band gap (Eg) of 3.18 eV, revealing a reduction in band gap energy.

ZnO@BC has a narrow band gap. On the one hand, the possible formation of new energy states in the hybrid ZnO@BC composite samples induced by Zn–O–C bonds formed due to an interaction of ZnO with the carbon content of ZnO@BC [58]. On the other hand, there are certain narrow band gap substances in the prepared biomass carbon, with reduced energy required for the electrons of ZnO to transition from the valence band to the conduction band. It can be observed that, if the spectral response range of ZnO@BC is extended and the utilization rate of light is improved, then the photocatalytic performance could be improved.

#### 3.3.2. Photoluminescence Analysis

**Figure 7:**
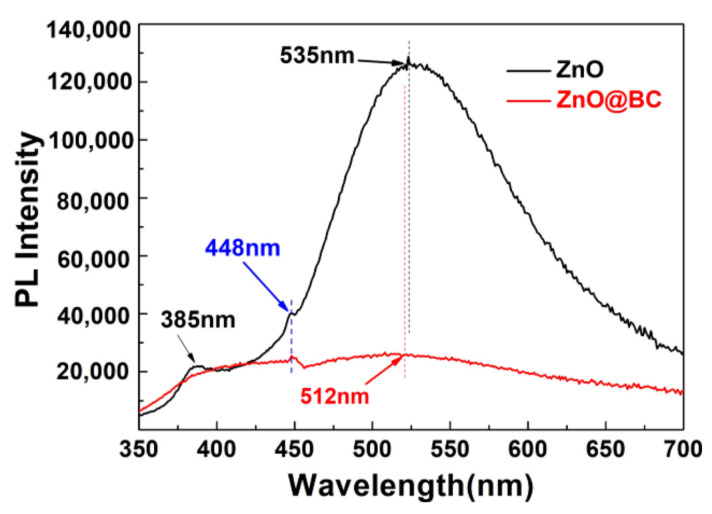
PL spectra of ZnO@BC.

As is known, electrons transition from the valence band to the conduction band and leave holes in the valence band under the excitation of light, and the combination of electrons and holes lead to photoluminescence. The photoluminescence spectroscopy can be applied to analyze surface vacancies, defect energy levels, and charge transfer of photocatalysts [59]. Usually, the peak of photoluminescence is weaker, the recombination rate of an electron–hole is lower, and photocatalytic activity is higher. It can be observed that the emission at 385 nm (3.21 eV) in the PL spectra of ZnO (Figure 7) can be ascribed to the near-band-edge (NBE) emission due to their combination of electrons from the minimum conduction band with holes of the valence band of the semiconducting ZnO. The following emission at 448 nm (2.76 eV) can be attributed to the oxygen vacancies (Vo)→valence band (VB) transition. The last emission at 535 nm (2.29 eV) is attributable to the conduction band (CB)→ oxygen antisites (O_Zn_). Compared with the PL spectra of ZnO, that of ZnO@BC shows a significant decrease and only one peak at 448 nm observed in the spectra, indicating that the biochar and surface oxygen defects quench the fluorescence from ZnO. In the previous studies, it had been demonstrated that the quenching of fluorescence could improve the charge transfer and reduce the recombination rate of electron–hole pair [60,61]. Therefore, it could be indicated that ZnO@BC has a higher photocatalytic activity than ZnO.

### 3.4. Photocatalysis Performance of MG Using ZnO@BC

**Figure 8:**
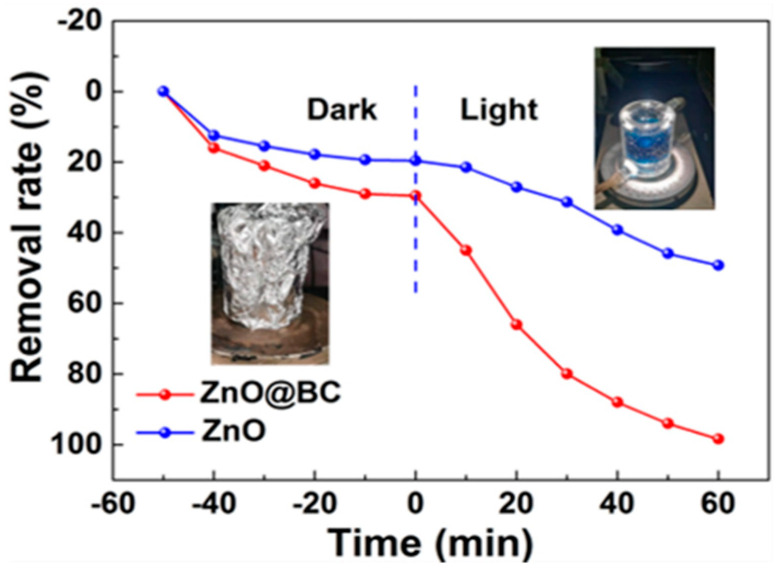
The removal rate of MG on ZnO@BC under visible light irradiation.

**Figure 9:**
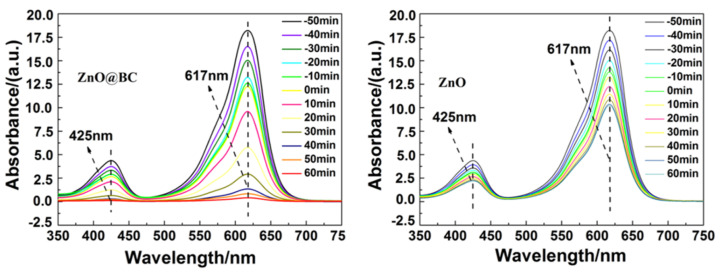
UV–vis absorption spectra of malachite green (MG) solution.

The photocatalytic activity of ZnO@BC and ZnO was evaluated in malachite green (MG) dye degradation, as shown in Figure 8. Before irradiation, the adsorption equilibrium of MG using photocatalysts needs to be established. It is observed that the removal rates of MG on ZnO@BC and ZnO are 29.5% and 19.58%, respectively, at adsorption equilibrium. After 60 min visible light irradiation, the photocatalytic degradation efficiencies of MG using ZnO@BC and ZnO are 68.88% and 29.64%, respectively, and the total removal rates of MG are 98.38% and 49.22%, respectively. As shown in Figure 9, the intensity of the absorption peaks of MG decreases with irradiation time, indicating that MG was degraded by photocatalysts, and the chromophoric groups of MG were gradually destroyed and degraded into smaller size molecules.

### 3.5. Active Species Analysis

**Figure 10:**
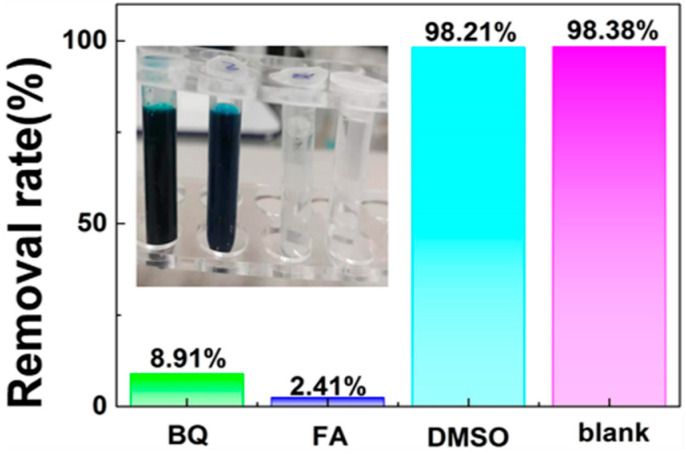
Effects of BQ, FA and DMSO on the photocatalytic degradation rates of MG on ZnO@BC under visible light irradiation.

The active species in the photocatalytic degradation process were detected by scavenger trapping experiments. Dimethyl sulfoxide (DMSO), formic acid (FA) and *p*-benzoquinone (BQ) were used as scavengers for •OH, H^+^ and •O^2−^, respectively. It can be demonstrated that the addition of BQ and FA could obviously reduce the removal rate of MG using ZnO@BC, as illustrated from Figure 10, suggesting that H^+^ and •O^2−^ play a vital role in the effective degradation of MG using ZnO@BC.

### 3.6. Photocatalytic Degradation Products of MG Analysis

**Figure 11:**
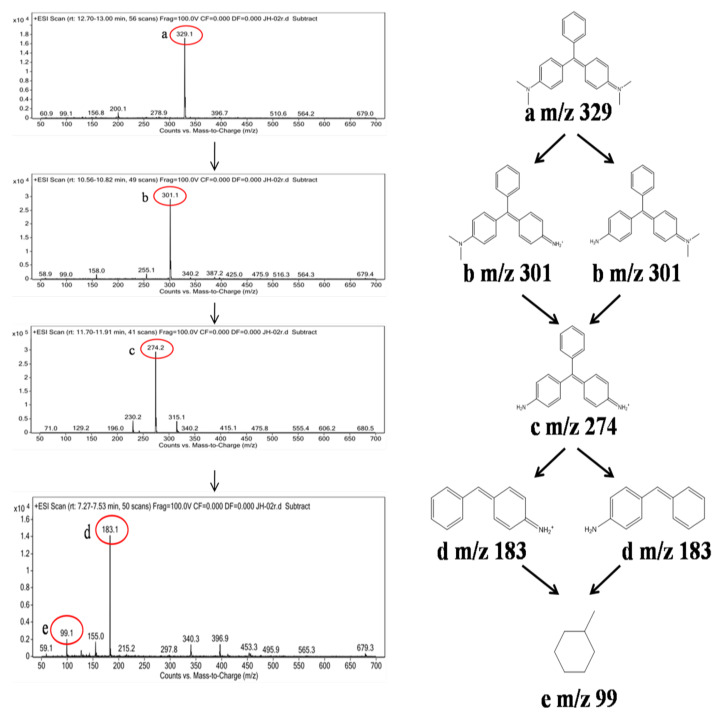
LC–MS analysis of the degradation pathway of MG by ZnO@BC.

LC–ESI–MS was employed to analyze the degradation intermediate products of MG using ZnO@BC. As shown in Figure 11, it can be found that product ‘a’ at the m/z value of 329 is MG; product ‘b’ at the *m*/*z* value of 301, containing two molecular formulas, is due to the removal of two methyl groups;, product ‘c’ at the *m*/*z* value of 274 is due to the removal of the other two methyl groups; product ‘d’ at the m/z value of 183 is due to the deprivation of one amino group and one benzene ring; and product ‘e’ at the *m*/*z* value of 99 is the result of the bonding bonds of the two benzene rings being further broken and the double bonds of the benzene ring breaking down into single bonds. Based on these degradation intermediate products, in which MG underwent demethylation, deamination, conjugate structure destruction and benzene ring structure destruction, ZnO@BC is suggested to degrade MG via a possible photocatalytic degradation pathway. Thus, during photocatalytic degradation, the reactive species very likely first attack the methyl group and amine group, which is responsible for the color of MG, and then the central carbon atom and benzene ring, and finally proceed with further deep degradation.

## 4. Discussion

As this article has demonstrated, the as-prepared photocatalyst (ZnO@BC) has an abundant porous structure and larger specific surface area, which can provide more adsorption sites, contributing to the photocatalytic degradation of MG. The XRD results show that ZnO nanoparticles have been successfully synthesized and analyzed by SEM and TEM images. While ZnO has a nanometer size and good dispersibility, it is beneficial to increase the transport rate of photogenerated carriers and to prevent the recombination of electrons and holes in ZnO@BC [62]. FT-IR analysis shows that ZnO@BC has a large number of hydroxyl groups, which is easily combined with photogenerated holes that can effectively prevent the recombination of electron–hole pairs. It can be calculated that the band gap (Eg) of ZnO@BC is 2.59 eV using UV-vis DRS analysis, improving the utilization efficiency of light. Therefore, ZnO@BC exhibits a higher catalytic degradation performance for MG, with a total removal rate of up to 98.38%. XPS and PL analysis reveal that oxygen vacancy plays a key role in the photocatalytic degradation of MG using ZnO@BC. This not only becomes the capture center of photogenerated electrons, inhibiting the recombination of photogenerated electrons and holes, but also promotes the adsorption of oxygen on ZnO@BC, accelerates the reaction of photogenerated electrons and adsorbed oxygen, promotes the generation of active substances, and then improves the decolorization and degradation of MG. Moreover, in the degradation of MG, using ZnO@BC, H^+^ and •O^2−^ as the major reactive species could degrade MG by demethylation, deamination, conjugate structure and benzene ring structure destruction.

## 5. Conclusions

In this paper, ZnO nanoparticles, synthesized from *S. alterniflora* extract and mixed with *S. alterniflora*, were prepared into a ZnO photocatalyst loaded on biochar (ZnO@BC) by a one-step carbonization method. The as-prepared sample has a higher photocatalytic degradation efficiency, and the total removal rate of MG using ZnO@BC is up to 98.38%. Oxygen vacancy is considered a critical factor in improving the photocatalytic activity, which could produce more reactive species (H^+^ and •O^2−^) to degrade MG by demethylation, deamination, conjugate structure and benzene ring structure destruction. Consequently, *S. alterniflora* has a huge potential in high-value utilization and wastewater treatment.

## Data Availability

The data is included in the main text, raw data are available upon request.
